# Supplementary material for: High Diversity and Low Coinfections of Pathogens in Ticks from Ruminants in Pakistan
Source: Microorganisms. 2025 May 30;13(6):1276. doi: 10.3390/microorganisms13061276 (PMC12195261; doi:10.3390/microorganisms13061276)
Supplement: Supplementary file 1 [file microorganisms-13-01276-s001.zip › microorganisms-3639845-supplementary.pdf]

Table S1. Geographical information of sampling sites and associated host species in Pakistan.

Province	District	Town	Longitude	Latitude	Host species
Khyber Pakhtunkhwa	Swabi	Maneri Payan	34.1445° N	72.4909° E	Cattle
	Swabi	Swabi	34.1270° N	72.4741° E	Cattle
	Swabi	Saleem khan khel	34.1664° N	72.4621° E	Goat/Sheep/Cattle
	Swabi	Batakara	34.0338° N	72.6003° E	Cattle
	Swabi	Jhanda	34.1523° N	72.5829° E	Cattle
	Swabi	Beka	33.9925° N	72.3483° E	Cattle
	Swabi	Panjman	34.1749° N	72.5782° E	Goat/Sheep
	Buner	Totalai	34.1923° N	72.4990° E	Cattle
	Buner	Dagai	34.1892° N	72.5043° E	Cattle
	Buner	Kas koruna	34.5083° N	72.3835° E	Cattle
	Buner	Chinglai	34.3227° N	72.5116° E	Goat
	Buner	Sawawai	34.2897° N	72.5160° E	Goat
	Buner	Daggar	34.5107° N	72.4835° E	Cattle
	Swat	Islampur	34.7211° N	72.3652° E	Goat/Sheep
	Swat	Bagh	35.0537° N	72.4734° E	Cattle
	Swat	Tangobanda	34.6088° N	72.5984° E	Goat/Sheep
	Swat	Kokarai	34.7387° N	72.4251° E	Goat/Sheep/Cattle
	Swat	Guligram	34.7330° N	72.3330° E	Goat/Sheep
	Swat	Bara bandai	34.8245° N	72.3947° E	Goat/Sheep
	Swat	Marghzar	34.6638° N	72.3481° E	Cattle
	Swat	Ingaraderai	34.8096° N	72.3577° E	Goat/Sheep

Table S2. List of primers used to amplify target genes of tick-borne pathogens.

Pathogen	Target gene	Primers' name	Primer sequence (5'-3')	Annealing temperature	Amplicon size
Anaplasma	16S rRNA	OUT1	TTGAGAGTTTGATCCTGGCTCAGAACG	50 °C	1500 bp
		3-17	TAAGGTGGTAATCCAGC		
		OUT1	TTGAGAGTTTGATCCTGGCTCAGAACG	55 °C	660 bp
		OUT2	CACCTCTACACTAGGAATTCCGCTATC		
	groEL	gro-1F	GAAGATGCWGTWGGWTGTACKGC	50 °C	730 bp
		gro-1R	AGMGCTTCWCCTTCWACRTCYTC		
		gro-1F	ATTACTCAGAGTGCTTCTCARTG	52 °C	364 bp
		gro-2R	TGCATACCRTCAGTYTTTTCAAC		
	gltA	gltA-1F	GCGATTTTAGAGTGYGGAGATTG	50 °C	1077 bp
		gltA-1R	TACAATACCGGAGTAAAAGTCAA		
		gltA-2F	GGGTTCMTGTCYACTGCTGCGTG	52 °C	793 bp
		gltA-2R	TTGGATCGTARTTCTTGTAGACC		
Rickettsia	*OmpA*	70F	ATGGCGAATATTTCTCAAAAA	50 °C	630 bp
		602R	AGTGCAGCATTCGCTCCCCCT		
		70F	ATGGCGAATATTTCTCAAAAA	50 °C	530 bp
		701R	GTTCCGTTAATGGCAGCATCT		
	17kDa	17K3	GCTTTACAAAATTCTAAAACCATATA	52 °C	500 bp
		17k5	TGTCTATCAATTCACAACTTGCC		
		17kDa-2F	ATTGTCCGTCAGGTTGGC	52°C	395 bp
		17kDa-2R	CGGGCGGTATGAATAAGC		
	gltA	RgltA-1F	ATGACCAATGAAAATAATAAT	50°C	1100 bp
		RgltA-1R	CTTATACTCTCTATGTACA		
		RgltA-2F	GGGGACCTGCTCACGGCGG	52°C	381 bp
		RgltA-2R	ATTGCAAAAAGTACAGTGAACA		
Babesia	18S rRNA	piroA	AATTACCCAATCCTGACACAG	58°C	373 bp
		piroB	TAAATACGAATGCCCCCAA		
Borrelia	5S-23S	23S3	CGACCTTCTTCGCCTTAAAGC	55°C	412 bp
		23Sa	TAAGCTGACTAATACTAATTACCC		
		23S5	CTGCGAGTTCGCGGGAGA	59°C	253 bp
		23S6	TCCTAGGCATTCACCATA		

Table S3. GenBank accession numbers of *Anaplasma* sequences obtained in this study.

Sample ID	Tick species	Host	District	Pathogen	GenBank accession number		
					**16S rRNA**	***groEL***	***gltA***
TIGMIC-001	*Hy. marginatum*	Goat/Sheep	Swabi	*Anaplasma ovis*	PQ817906	PV026429	-
TIGMIC-002	*Hy. marginatum*	Goat/Sheep	Swabi	*Anaplasma ovis*	PQ817907	PV026430	PV026336
TIGMIC-003	*Hy. marginatum*	Goat/Sheep	Swabi	*Anaplasma ovis*	PQ817908	PV026432	PV026338
TIGMIC-004	*Hy. marginatum*	Goat/Sheep	Swabi	*Anaplasma ovis*	PQ817909	PV026431	PV026337
TIGMIC-005	*Hy. marginatum*	Goat/Sheep	Swabi	*Anaplasma ovis*	PQ817910	PV026433	-
TIGMIC-006	*Hy. marginatum*	Goat/Sheep	Swabi	*Anaplasma ovis*	PQ817911	PV026434	PV026339
TIGMIC-007	*Hy. marginatum*	Goat/Sheep	Swabi	*Anaplasma ovis*	PQ817912	PV026435	PV026340
TIGMIC-008	*Hy. marginatum*	Goat/Sheep	Swabi	*Anaplasma ovis*	PQ817913	PV026436	PV026341
TIGMIC-009	*Hy. marginatum*	Goat/Sheep	Swabi	*Anaplasma ovis*	PQ817914	PV026437	PV026342
TIGMIC-010	*Hy. marginatum*	Goat/Sheep	Swabi	*Anaplasma ovis*	PQ817915	PV026438	PV026343
TIGMIC-011	*Hy. marginatum*	Goat/Sheep	Swabi	*Anaplasma ovis*	PQ817916	PV026439	PV026344
TIGMIC-012	*Hy. marginatum*	Goat/Sheep	Swabi	*Anaplasma ovis*	PQ817917	PV026440	PV026345
TIGMIC-013	*Hy. marginatum*	Goat/Sheep	Swabi	*Anaplasma ovis*	PQ817918	PV026442	PV026347
TIGMIC-014	*Hy. marginatum*	Goat/Sheep	Swabi	*Anaplasma ovis*	PQ817919	PV026443	PV026348
TIGMIC-015	*Hy. marginatum*	Goat/Sheep	Swabi	*Anaplasma ovis*	PQ817920	PV026444	PV026349
TIGMIC-016	*Hy. marginatum*	Goat/Sheep	Swabi	*Anaplasma ovis*	PQ817921	PV026445	PV026350
TIGMIC-017	*Hy. marginatum*	Goat/Sheep	Swabi	*Anaplasma ovis*	PQ817922	PV026447	-
TIGMIC-018	*Hy. marginatum*	Goat/Sheep	Swabi	*Anaplasma ovis*	PQ817923	PV026448	PV026352
TIGMIC-019	*Hy. marginatum*	Goat/Sheep	Swabi	*Anaplasma ovis*	PQ817924	PV026449	PV026353
TIGMIC-020	*Hy. marginatum*	Goat/Sheep	Swabi	*Anaplasma ovis*	PQ817925	PV026450	PV026354
TIGMIC-021	*Hy. marginatum*	Goat/Sheep	Swabi	*Anaplasma ovis*	PQ817926	PV026452	PV026356
TIGMIC-022	*Hy. marginatum*	Goat/Sheep	Swabi	*Anaplasma ovis*	PQ817927	PV026454	PV026358
TIGMIC-023	*Hy. marginatum*	Goat/Sheep	Swabi	*Anaplasma ovis*	PQ817928	PV026455	PV026359

TIGMIC-024	*Hy. marginatum*	Goat/Sheep	Swabi	*Anaplasma ovis*	PQ817929	PV026456	PV026360
TIGMIC-025	*Hy. marginatum*	Goat/Sheep	Swabi	*Anaplasma ovis*	PQ817930	PV026457	PV026361
TIGMIC-026	*Hy. marginatum*	Goat/Sheep	Swabi	*Anaplasma ovis*	PQ817931	PV026459	PV026363
TIGMIC-027	*Hy. marginatum*	Goat/Sheep	Swabi	*Anaplasma ovis*	PQ817932	PV026460	PV026364
TIGMIC-028	*Hy. marginatum*	Goat/Sheep	Swabi	*Anaplasma ovis*	PQ817933	PV026461	-
TIGMIC-029	*Hy. marginatum*	Goat/Sheep	Swabi	*Anaplasma ovis*	PQ817934	PV026462	PV026365
TIGMIC-030	*Hy. marginatum*	Goat/Sheep	Swabi	*Anaplasma ovis*	PQ817935	PV026463	-
TIGMIC-031	*Hy. marginatum*	Goat/Sheep	Swabi	*Anaplasma ovis*	PQ817936	PV026464	PV026366
TIGMIC-032	*Hy. marginatum*	Goat/Sheep	Swabi	*Anaplasma ovis*	PQ817937	PV026465	PV026367
TIGMIC-033	*Hy. marginatum*	Goat/Sheep	Swabi	*Anaplasma ovis*	PQ817938	PV026466	PV026368
TIGMIC-034	*Hy. marginatum*	Goat/Sheep	Swabi	*Anaplasma ovis*	PQ817939	PV026467	-
TIGMIC-035	*Hae. bispinosa*	Goat/Sheep	Swabi	*Anaplasma ovis*	PQ817901	PV026422	-
TIGMIC-036	*Hae. bispinosa*	Goat/Sheep	Swabi	*Anaplasma ovis*	PQ817902	PV026423	PV026332
TIGMIC-037	*Hae. bispinosa*	Goat/Sheep	Swabi	*Anaplasma ovis*	PQ817903	PV026424	-
TIGMIC-038	*Hae. bispinosa*	Goat/Sheep	Swabi	*Anaplasma ovis*	PQ817904	PV026425	-
TIGMIC-039	*Hae. bispinosa*	Goat/Sheep	Swabi	*Anaplasma ovis*	PQ817905	PV026426	PV026333
TIGMIC-040	*R. sanguineus*	Goat/Sheep	Swabi	*Anaplasma ovis*	PQ817941	PV026468	PV026369
TIGMIC-041	*R. sanguineus*	Goat/Sheep	Swabi	*Anaplasma ovis*	PQ817942	PV026469	-
TIGMIC-042	*R. sanguineus*	Goat/Sheep	Swabi	*Anaplasma ovis*	PQ817943	PV026470	PV026370
TIGMIC-043	*R. haemaphysaloides*	Sheep	Buner	*Anaplasma ovis*	PQ817940	PV026471	PV026331
TIGMIC-044	*R. microplus*	Cattle	Buner	*Anaplasma marginale*	PQ817850	PV026371	-
TIGMIC-045	*R. microplus*	Cattle	Buner	*Anaplasma marginale*	PQ817851	PV026372	-
TIGMIC-046	*R. microplus*	Cattle	Buner	*Anaplasma marginale*	PQ817852	PV026373	-
TIGMIC-047	*R. microplus*	Cattle	Buner	*Anaplasma marginale*	PQ817853	PV026374	-
TIGMIC-048	*R. microplus*	Cattle	Buner	*Anaplasma marginale*	PQ817854	PV026375	-
TIGMIC-049	*R. microplus*	Cattle	Buner	*Anaplasma marginale*	PQ817855	PV026376	-

TIGMIC-050	*R. microplus*	Cattle	Swat	*Anaplasma marginale*	PQ817856	PV026421	-
TIGMIC-051	*R. microplus*	Cattle	Swat	*Anaplasma marginale*	PQ817857	PV026377	PV026312
TIGMIC-052	*R. microplus*	Cattle	Swat	*Anaplasma marginale*	PQ817858	PV026378	PV026313
TIGMIC-053	*R. microplus*	Cattle	Swat	*Anaplasma marginale*	PQ817859	PV026379	PV026314
TIGMIC-054	*R. microplus*	Cattle	Swat	*Anaplasma marginale*	PQ817860	PV026380	PV026315
TIGMIC-055	*R. microplus*	Cattle	Swat	*Anaplasma marginale*	PQ817861	PV026381	PV026316
TIGMIC-056	*R. microplus*	Cattle	Swat	*Anaplasma marginale*	PQ817862	PV026382	PV026317
TIGMIC-057	*R. microplus*	Cattle	Swat	*Anaplasma marginale*	PQ817863	PV026383	PV026318
TIGMIC-058	*R. microplus*	Cattle	Swat	*Anaplasma marginale*	PQ817864	PV026384	-
TIGMIC-059	*R. microplus*	Cattle	Swat	*Anaplasma marginale*	PQ817865	PV026385	PV026319
TIGMIC-060	*R. microplus*	Cattle	Swat	*Anaplasma marginale*	PQ817866	PV026386	PV026320
TIGMIC-061	*R. microplus*	Cattle	Swat	*Anaplasma marginale*	PQ817867	PV026387	-
TIGMIC-062	*R. microplus*	Cattle	Swat	*Anaplasma marginale*	PQ817868	PV026388	-
TIGMIC-063	*R. microplus*	Cattle	Swat	*Anaplasma marginale*	PQ817869	PV026389	-
TIGMIC-064	*R. microplus*	Cattle	Swat	*Anaplasma marginale*	PQ817870	PV026390	-
TIGMIC-065	*R. microplus*	Cattle	Swat	*Anaplasma marginale*	PQ817871	PV026391	-
TIGMIC-066	*R. microplus*	Cattle	Swat	*Anaplasma marginale*	PQ817872	PV026392	-
TIGMIC-067	*R. microplus*	Cattle	Swat	*Anaplasma marginale*	PQ817873	PV026393	PV026321
TIGMIC-068	*R. microplus*	Cattle	Swat	*Anaplasma marginale*	PQ817874	PV026394	-
TIGMIC-069	*R. microplus*	Cattle	Swat	*Anaplasma marginale*	PQ817875	PV026395	PV026322
TIGMIC-070	*R. microplus*	Cattle	Swat	*Anaplasma marginale*	PQ817876	PV026396	PV026323
TIGMIC-071	*R. microplus*	Cattle	Swat	*Anaplasma marginale*	PQ817877	PV026397	-
TIGMIC-072	*R. microplus*	Cattle	Swat	*Anaplasma marginale*	PQ817878	PV026398	PV026324
TIGMIC-073	*R. microplus*	Cattle	Swat	*Anaplasma marginale*	PQ817879	PV026399	PV026325
TIGMIC-074	*R. microplus*	Cattle	Swat	*Anaplasma marginale*	PQ817880	PV026400	PV026326
TIGMIC-075	*R. microplus*	Cattle	Swat	*Anaplasma marginale*	PQ817881	PV026401	PV026327

TIGMIC-076	*R. microplus*	Cattle	Swat	*Anaplasma marginale*	PQ817882	PV026402	-
TIGMIC-077	*R. microplus*	Cattle	Swat	*Anaplasma marginale*	PQ817883	PV026403	-
TIGMIC-078	*R. microplus*	Cattle	Swat	*Anaplasma marginale*	PQ817884	PV026404	-
TIGMIC-079	*R. microplus*	Cattle	Swat	*Anaplasma marginale*	PQ817885	PV026405	-
TIGMIC-080	*R. microplus*	Cattle	Swat	*Anaplasma marginale*	PQ817886	PV026406	-
TIGMIC-081	*R. microplus*	Cattle	Swat	*Anaplasma marginale*	PQ817887	PV026407	-
TIGMIC-082	*R. microplus*	Cattle	Swat	*Anaplasma marginale*	PQ817888	PV026408	-
TIGMIC-083	*R. microplus*	Cattle	Swat	*Anaplasma marginale*	PQ817889	PV026409	-
TIGMIC-084	*R. microplus*	Cattle	Swat	*Anaplasma marginale*	PQ817890	PV026410	-
TIGMIC-085	*R. microplus*	Cattle	Swat	*Anaplasma marginale*	PQ817891	PV026411	-
TIGMIC-086	*R. microplus*	Cattle	Swat	*Anaplasma marginale*	PQ817892	PV026412	-
TIGMIC-087	*R. microplus*	Cattle	Swat	*Anaplasma marginale*	PQ817893	PV026413	-
TIGMIC-088	*R. microplus*	Cattle	Swat	*Anaplasma marginale*	PQ817894	PV026414	-
TIGMIC-089	*R. microplus*	Cattle	Swat	*Anaplasma marginale*	PQ817895	PV026415	-
TIGMIC-090	*R. microplus*	Cattle	Swat	*Anaplasma marginale*	PQ817896	PV026416	PV026328
TIGMIC-091	*R. microplus*	Cattle	Swat	*Anaplasma marginale*	PQ817897	PV026417	PV026329
TIGMIC-092	*R. microplus*	Cattle	Swat	*Anaplasma marginale*	PQ817898	PV026418	PV026330
TIGMIC-093	*R. microplus*	Cattle	Swat	*Anaplasma marginale*	PQ817899	PV026419	-
TIGMIC-094	*R. microplus*	Cattle	Swat	*Anaplasma marginale*	PQ817900	PV026420	-
TIGMIC-095	*Hy. marginatum*	Goat/Sheep	Swabi	*Anaplasma ovis*	-	PV026427	PV026334
TIGMIC-096	*Hy. marginatum*	Goat/Sheep	Swabi	*Anaplasma ovis*	-	PV026428	PV026335
TIGMIC-097	*Hy. marginatum*	Goat/Sheep	Swabi	*Anaplasma ovis*	-	PV026441	PV026346
TIGMIC-098	*Hy. marginatum*	Goat/Sheep	Swabi	*Anaplasma ovis*	-	PV026446	PV026351
TIGMIC-099	*Hy. marginatum*	Goat/Sheep	Swabi	*Anaplasma ovis*	-	PV026451	PV026355
TIGMIC-100	*Hy. marginatum*	Goat/Sheep	Swabi	*Anaplasma ovis*	-	PV026453	PV026357
TIGMIC-101	*Hy. marginatum*	Goat/Sheep	Swabi	*Anaplasma ovis*	-	PV026458	PV026362

Table S4. Nucleotide identity of *Anaplasma marginale* positive sequences based on BLAST analysis of the *gltA* gene.

Sample ID	Gene	Best Match Species	GenBank Accession No.	Sequence Identity (%)	Sequence Coverage (%)	E-value	Host	Country
TIGMIC-051	*gltA*	*Anaplasma marginale*	MT722114.1	100	90	0.0	*Amblyomma cajennense*	Colombia
TIGMIC-052	*gltA*	*Anaplasma marginale*	AF304140.1	100	100	0.0	-	Florida
TIGMIC-053	*gltA*	*Anaplasma marginale*	MT722114.1	100	90	0.0	*Amblyomma cajennense*	Colombia
TIGMIC-054	*gltA*	*Anaplasma marginale*	AF304140.1	100	100	0.0	-	Florida
TIGMIC-055	*gltA*	*Anaplasma marginale*	MT722098.1	100	96	0.0	*Rhipicephalus microplus*	Colombia
TIGMIC-056	*gltA*	*Anaplasma marginale*	CP006847.1	100	100	0.0	Cattle	Australia
TIGMIC-057	*gltA*	*Anaplasma marginale*	AF304140.1	100	100	0.0	-	Florida
TIGMIC-059	*gltA*	*Anaplasma marginale*	AF304140.1	100	100	0.0	-	Florida
TIGMIC-060	*gltA*	*Anaplasma marginale*	AF304140.1	100	100	0.0	-	Florida
TIGMIC-067	*gltA*	*Anaplasma marginale*	CP006847.1	100	100	0.0	Cattle	Australia
TIGMIC-069	*gltA*	*Anaplasma marginale*	CP006847.1	100	100	0.0	Cattle	Australia
TIGMIC-070	*gltA*	*Anaplasma marginale*	CP006847.1	100	100	0.0	Cattle	Australia
TIGMIC-072	*gltA*	*Anaplasma marginale*	AF304140.1	100	100	0.0	-	Florida
TIGMIC-073	*gltA*	*Anaplasma marginale*	AF304140.1	100	100	0.0	-	Florida
TIGMIC-074	*gltA*	*Anaplasma marginale*	AF304140.1	100	100	0.0	-	Florida
TIGMIC-075	*gltA*	*Anaplasma marginale*	AF304140.1	100	100	0.0	-	Florida
TIGMIC-090	*gltA*	*Anaplasma marginale*	AF304140.1	100	100	0.0	-	Florida
TIGMIC-091	*gltA*	*Anaplasma marginale*	AF304140.1	100	100	0.0	-	Florida
TIGMIC-092	*gltA*	*Anaplasma marginale*	AF304140.1	100	100	0.0	-	Florida

Table S5. Association between tick species and *Anaplasma* based on 2 × n contingency tables, with Fisher's exact test results.

Pathogen	Tick Species	Positive	Negative	Total	Fisher's Exact Test p-value
Anaplasma ovis	*Hy. marginatum*	41	116	157	$p < 0.0001$
	Hae. bispinosa	5	44	49	
	R. sanguineus	3	14	17	
	R. haemaphysaloides	1	12	13	
	R. microplus	0	753	753	
Anaplasma marginale	*Hy. marginatum*	0	157	157	$p < 0.0001$
	Hae. bispinosa	0	49	49	
	R. sanguineus	0	17	17	
	R. haemaphysaloides	0	13	13	
	R. microplus	51	702	753	

Table S6. GenBank accession numbers of *Ehrlichia* sequences obtained in this study.

Sample ID	Tick species	Host	District	Pathogen	GenBank accession number	
					**16S rRNA**	***groEL***
TIGMIC-178	*R. microplus*	Cattle	Swabi	*Ehrlichia minasensis*	PQ824685	PV026476
TIGMIC-179	*Hy. marginatum*	Goat/Sheep	Swabi	*Candidatus* Ehrlichia hyalommae	PQ824675	PV026477
TIGMIC-180	*Hy. marginatum*	Goat/Sheep	Swabi	*Candidatus* Ehrlichia hyalommae	PQ824676	PV026478
TIGMIC-181	*Hy. marginatum*	Goat/Sheep	Swabi	*Candidatus* Ehrlichia hyalommae	PQ824677	PV026479
TIGMIC-182	*R. microplus*	Cattle	Swat	*Candidatus* Ehrlichia rhipicephalis	PQ824678	PV026475
TIGMIC-183	*R. microplus*	Cattle	Swat	*Candidatus* Ehrlichia rhipicephalis	PQ824679	PV026481
TIGMIC-184	*R. microplus*	Cattle	Swat	*Candidatus* Ehrlichia rhipicephalis	PQ824680	PV026474
TIGMIC-185	*R. microplus*	Cattle	Buner	*Candidatus* Ehrlichia rhipicephalis	PQ824681	PV026480
TIGMIC-186	*R. microplus*	Cattle	Swat	*Candidatus* Ehrlichia rhipicephalis	PQ824682	PV026482
TIGMIC-187	*R. microplus*	Cattle	Swabi	*Candidatus* Ehrlichia rhipicephalis	PQ824683	PV026473
TIGMIC-188	*R. microplus*	Cattle	Swabi	*Candidatus* Ehrlichia rhipicephalis	PQ824684	PV026472

Table S7. Association between tick species and *Ehrlichia* based on 2 × n contingency tables, with Fisher's exact test results.

Pathogen	Tick Species	Positive	Negative	Total	Fisher's Exact Test p-value
Ehrlichia minasensis	*Hy. marginatum*	0	157	157	$p = 1.00000$
	Hae. bispinosa	0	49	49	
	R. sanguineus	0	17	17	
	R. haemaphysaloides	0	13	13	
	R. microplus	1	752	753	
Candidatus E. hyalommae	*Hy. marginatum*	3	154	157	$p < 0.05$
	Hae. bispinosa	0	49	49	
	R. sanguineus	0	17	17	
	R. haemaphysaloides	0	13	13	
	R. microplus	0	753	753	
Candidatus E. rhipicephalis	*Hy. marginatum*	0	157	157	$p = 0.7832$
	Hae. bispinosa	0	49	49	
	R. sanguineus	0	17	17	
	R. haemaphysaloides	0	13	13	
	R. microplus	7	746	753	

Table S8. GenBank accession numbers of *Rickettsia* sequences obtained in this study.

Sample ID	Tick species	Host	District	Pathogen	GenBank accession number		
					OmpA	***17KDa***	***gltA***
TIGMIC-004	*Hy. marginatum*	Goat/Sheep	Swabi	*Candidatus* Rickettsia pakistanensis	PV026493	PV026509	PV026485
TIGMIC-177	*Hy. marginatum*	Goat/Sheep	Swabi	*Candidatus* Rickettsia pakistanensis	PV026494	PV026504	PV026487
TIGMIC-189	*Hy. marginatum*	Goat/Sheep	Swabi	*Candidatus* Rickettsia pakistanensis	PV026495	PV026508	PV026488
TIGMIC-190	*Hy. marginatum*	Goat/Sheep	Swabi	*Candidatus* Rickettsia pakistanensis	PV026496	PV026507	PV026483
TIGMIC-191	*Hy. marginatum*	Goat/Sheep	Swabi	*Candidatus* Rickettsia pakistanensis	PV026497	PV026505	PV026484
TIGMIC-192	*Hy. marginatum*	Goat/Sheep	Swabi	*Candidatus* Rickettsia pakistanensis	PV026498	-	PV026489
TIGMIC-193	*R. sanguineus*	Goat/Sheep	Swabi	*Rickettsia massiliae*	PV026499	PV026512	-
TIGMIC-194	*R. sanguineus*	Goat/Sheep	Swabi	*Rickettsia massiliae*	PV026500	PV026514	-
TIGMIC-195	*R. sanguineus*	Goat/Sheep	Swabi	*Rickettsia massiliae*	PV026501	PV026515	PV026490
TIGMIC-196	*R. sanguineus*	Goat/Sheep	Swabi	*Rickettsia massiliae*	PV026502	PV026511	-
TIGMIC-197	*R. microplus*	Sheep	Buner	*Rickettsia felis*	PV026503	PV026311	-
TIGMIC-198	*R. sanguineus*	Goat/Sheep	Swabi	*Rickettsia massiliae*	-	PV026513	PV026491
TIGMIC-199	*R. microplus*	Cattle	Buner	*Rickettsia massiliae*	-	PV026510	PV026492
TIGMIC-200	*Hy. marginatum*	Goat/Sheep	Swabi	*Candidatus* Rickettsia pakistanensis	-	PV026506	PV026486

Table S9. GenBank accession numbers of *Babesia* and *Theileria* sequences obtained in this study.

Sample ID	Tick species	Host	District	Pathogen	GenBank accession number
					18S rRNA
TIGMIC-055	*R. microplus*	Cattle	Swat	*Babesia bigemina*	PQ818397
TIGMIC-073	*R. microplus*	Cattle	Swat	*Babesia bigemina*	PQ818398
TIGMIC-112	*R. microplus*	Cattle	Buner	*Theileria orientalis*	PV346931
TIGMIC-118	*R. microplus*	Cattle	Buner	*Theileria orientalis*	PV346932
TIGMIC-108	*R. microplus*	Cattle	Buner	*Theileria orientalis*	PV346933
TIGMIC-104	*R. microplus*	Cattle	Buner	*Theileria orientalis*	PV346934
TIGMIC-126	*R. microplus*	Cattle	Swat	*Theileria orientalis*	PV346935
TIGMIC-123	*R. microplus*	Cattle	Swat	*Theileria orientalis*	PV346936
TIGMIC-114	*R. microplus*	Cattle	Swat	*Theileria orientalis*	PV346937
TIGMIC-115	*R. microplus*	Cattle	Swat	*Theileria orientalis*	PV346938
TIGMIC-113	*R. microplus*	Cattle	Swat	*Theileria orientalis*	PV346939
TIGMIC-117	*R. microplus*	Cattle	Swat	*Theileria orientalis*	PV346940
TIGMIC-121	*R. microplus*	Cattle	Swat	*Theileria orientalis*	PV346941
TIGMIC-120	*R. microplus*	Cattle	Swat	*Theileria orientalis*	PV346942
TIGMIC-116	*R. microplus*	Cattle	Swat	*Theileria orientalis*	PV346943
TIGMIC-124	*R. microplus*	Cattle	Swat	*Theileria orientalis*	PV346944
TIGMIC-119	*R. microplus*	Cattle	Swat	*Theileria orientalis*	PV346945
TIGMIC-122	*R. microplus*	Cattle	Swat	*Theileria orientalis*	PV346946
TIGMIC-107	*R. microplus*	Cattle	Swat	*Theileria orientalis*	PV346947
TIGMIC-125	*R. microplus*	Cattle	Swabi	*Theileria orientalis*	PV346948
TIGMIC-111	*R. microplus*	Cattle	Swabi	*Theileria orientalis*	PV346949
TIGMIC-110	*R. microplus*	Cattle	Swabi	*Theileria orientalis*	PV346950
TIGMIC-105	*R. microplus*	Cattle	Swabi	*Theileria orientalis*	PV346951

TIGMIC-103	*R. microplus*	Cattle	Swabi	*Theileria orientalis*	PV346952
TIGMIC-109	*Hy. marginatum*	Goat/Sheep	Swabi	*Theileria orientalis*	PV346953
TIGMIC-106	*Hae. bispinosa*	Goat/Sheep	Swabi	*Theileria orientalis*	PV346954
TIGMIC-102	*R. microplus*	Sheep	Buner	*Theileria buffeli*	PV346955
TIGMIC-031	*Hy. marginatum*	Goat/Sheep	Swabi	*Theileria sinensis*	PV346956
TIGMIC-026	*Hy. marginatum*	Goat/Sheep	Swabi	*Theileria sinensis*	PV346957
TIGMIC-025	*Hy. marginatum*	Goat/Sheep	Swabi	*Theileria sinensis*	PV346958
TIGMIC-130	*Hae. bispinosa*	Goat/Sheep	Swabi	*Theileria sinensis*	PV346959
TIGMIC-036	*Hae. bispinosa*	Goat/Sheep	Swabi	*Theileria sinensis*	PV346960
TIGMIC-127	*Hae. bispinosa*	Goat/Sheep	Swabi	*Theileria sinensis*	PV346961
TIGMIC-131	*Hae. bispinosa*	Goat/Sheep	Swabi	*Theileria sinensis*	PV346962
TIGMIC-038	*Hae. bispinosa*	Goat/Sheep	Swabi	*Theileria sinensis*	PV346963
TIGMIC-129	*Hae. bispinosa*	Goat/Sheep	Swabi	*Theileria sinensis*	PV346964
TIGMIC-037	*Hae. bispinosa*	Goat/Sheep	Swabi	*Theileria sinensis*	PV346965
TIGMIC-128	*Hae. bispinosa*	Goat/Sheep	Swabi	*Theileria sinensis*	PV346966
TIGMIC-132	*Hae. bispinosa*	Goat/Sheep	Swabi	*Theileria sinensis*	PV346967
TIGMIC-166	*R. microplus*	Sheep	Buner	*Theileria luwenshuni*	PV346968
TIGMIC-176	*R. microplus*	Sheep	Buner	*Theileria luwenshuni*	PV346969
TIGMIC-167	*Hae. bispinosa*	Goat/Sheep	Swabi	*Theileria luwenshuni*	PV346970
TIGMIC-173	*Hae. bispinosa*	Goat/Sheep	Swabi	*Theileria luwenshuni*	PV346971
TIGMIC-169	*Hae. bispinosa*	Goat/Sheep	Swabi	*Theileria luwenshuni*	PV346972
TIGMIC-171	*Hae. bispinosa*	Goat/Sheep	Swabi	*Theileria luwenshuni*	PV346973
TIGMIC-172	*Hae. bispinosa*	Goat/Sheep	Swabi	*Theileria luwenshuni*	PV346974
TIGMIC-165	*Hy. marginatum*	Goat/Sheep	Swabi	*Theileria luwenshuni*	PV346975
TIGMIC-174	*Hy. marginatum*	Goat/Sheep	Swabi	*Theileria luwenshuni*	PV346976
TIGMIC-177	*Hy. marginatum*	Goat/Sheep	Swabi	*Theileria luwenshuni*	PV346977

TIGMIC-030	*Hy. marginatum*	Goat/Sheep	Swabi	*Theileria luwenshuni*	PV346978
TIGMIC-175	*Hy. marginatum*	Goat/Sheep	Swabi	*Theileria luwenshuni*	PV346979
TIGMIC-168	*Hy. marginatum*	Goat/Sheep	Swabi	*Theileria luwenshuni*	PV346980
TIGMIC-170	*Hy. marginatum*	Goat/Sheep	Swabi	*Theileria luwenshuni*	PV346981
TIGMIC-146	*R. microplus*	Cattle	Swabi	*Theileria annulata*	PV346982
TIGMIC-133	*R. microplus*	Cattle	Swabi	*Theileria annulata*	PV346983
TIGMIC-140	*R. microplus*	Cattle	Buner	*Theileria annulata*	PV346984
TIGMIC-149	*R. microplus*	Cattle	Buner	*Theileria annulata*	PV346985
TIGMIC-159	*R. microplus*	Cattle	Buner	*Theileria annulata*	PV346986
TIGMIC-148	*R. microplus*	Sheep	Buner	*Theileria annulata*	PV346987
TIGMIC-135	*R. microplus*	Cattle	Buner	*Theileria annulata*	PV346988
TIGMIC-155	*R. microplus*	Cattle	Buner	*Theileria annulata*	PV346989
TIGMIC-154	*R. microplus*	Cattle	Buner	*Theileria annulata*	PV346990
TIGMIC-082	*R. microplus*	Cattle	Swat	*Theileria annulata*	PV346991
TIGMIC-084	*R. microplus*	Cattle	Swat	*Theileria annulata*	PV346992
TIGMIC-086	*R. microplus*	Cattle	Swat	*Theileria annulata*	PV346993
TIGMIC-158	*R. microplus*	Cattle	Swat	*Theileria annulata*	PV346994
TIGMIC-087	*R. microplus*	Cattle	Swat	*Theileria annulata*	PV346995
TIGMIC-143	*R. microplus*	Cattle	Swat	*Theileria annulata*	PV346996
TIGMIC-145	*R. microplus*	Cattle	Swat	*Theileria annulata*	PV346997
TIGMIC-137	*R. microplus*	Cattle	Swat	*Theileria annulata*	PV346998
TIGMIC-162	*R. microplus*	Cattle	Swat	*Theileria annulata*	PV346999
TIGMIC-083	*R. microplus*	Cattle	Swat	*Theileria annulata*	PV347000
TIGMIC-151	*R. microplus*	Cattle	Swat	*Theileria annulata*	PV347001
TIGMIC-085	*R. microplus*	Cattle	Swat	*Theileria annulata*	PV347002
TIGMIC-161	*R. microplus*	Cattle	Swat	*Theileria annulata*	PV347003

TIGMIC-150	*R. microplus*	Cattle	Swat	*Theileria annulata*	PV347004
TIGMIC-147	*R. microplus*	Cattle	Swat	*Theileria annulata*	PV347005
TIGMIC-160	*R. microplus*	Cattle	Swat	*Theileria annulata*	PV347006
TIGMIC-077	*R. microplus*	Cattle	Swat	*Theileria annulata*	PV347007
TIGMIC-152	*R. microplus*	Cattle	Swat	*Theileria annulata*	PV347008
TIGMIC-142	*R. microplus*	Cattle	Swat	*Theileria annulata*	PV347009
TIGMIC-164	*R. microplus*	Cattle	Swat	*Theileria annulata*	PV347010
TIGMIC-139	*R. microplus*	Cattle	Swat	*Theileria annulata*	PV347011
TIGMIC-153	*R. microplus*	Cattle	Swat	*Theileria annulata*	PV347012
TIGMIC-163	*R. microplus*	Cattle	Swat	*Theileria annulata*	PV347013
TIGMIC-157	*R. microplus*	Cattle	Swat	*Theileria annulata*	PV347014
TIGMIC-070	*R. microplus*	Cattle	Swat	*Theileria annulata*	PV347015
TIGMIC-156	*R. microplus*	Cattle	Swat	*Theileria annulata*	PV347016
TIGMIC-136	*R. microplus*	Cattle	Swat	*Theileria annulata*	PV347017
TIGMIC-138	*R. microplus*	Cattle	Swat	*Theileria annulata*	PV347018
TIGMIC-141	*R. microplus*	Cattle	Swat	*Theileria annulata*	PV347019
TIGMIC-134	*R. microplus*	Cattle	Swat	*Theileria annulata*	PV347020
TIGMIC-066	*R. microplus*	Cattle	Swat	*Theileria annulata*	PV347021
TIGMIC-071	*R. microplus*	Cattle	Swat	*Theileria annulata*	PV347022
TIGMIC-080	*R. microplus*	Cattle	Swat	*Theileria annulata*	PV347023
TIGMIC-144	*R. microplus*	Cattle	Swat	*Theileria annulata*	PV347024

Table S10. 2×2 contingency table for co-infection of *A. marginale* and *T. annulata* in *Rhipicephalus microplus* (n = 753).

	T. annulata (+)	*T. annulata* (–)	Total
A. marginale (+)	11	40	51
A. marginale (–)	32	670	702
Total	43	710	753

$\chi^2 = 22.49$, df = 1, $p = 2.11 \times 10^{-6}$

Expected frequency of double infection = 2.91
